# Ruptured caesarean scar ectopic pregnancy: a diagnostic dilemma in a resource-limited setting

**DOI:** 10.1186/s13104-018-3389-3

**Published:** 2018-05-11

**Authors:** Atem Bethel Ajong, Bruno Kenfack, Valirie Ndip Agbor, Philip Nana Njotang

**Affiliations:** 1 Kekem District Hospital, Kekem, West Region, Cameroon; 2 Department of Biomedical Sciences, University of Dschang, Dschang, Cameroon; 3 Ibal Sub-Divisional Hospital, Oku, North-west Region, Cameroon; 4 Department of Obstetrics and Gynaecology, Faculty of Medicine and Biomedical Sciences, University of Yaoundé I, Yaoundé, Cameroon; 5 Obstetrics and Gynaecology Unit, Yaoundé Central Hospital, Yaoundé, Cameroon

**Keywords:** Caesarean scar pregnancy, Rupture, Ectopic pregnancy, Case report

## Abstract

**Background:**

Caesarean scar pregnancy (CSP) remains a very rare form of ectopic pregnancy associated with serious life threatening obstetric complications and even death in case of late diagnosis and treatment.

**Case presentation:**

We report a case of a ruptured caesarean scar pregnancy in a 29 year-old gravida 5, para 3 with a past obstetric history of two consecutive caesarean sections done 9 and 5 years ago respectively. The patient presented with intermittent lower abdominal pains on a 20 weeks gestation associated with mild epigastralgia and 2 previous episodes of mild pervaginal bleeding (2 and 1 months ago respectively before consultation) managed with injectable progesterone. Her evolution 4 h later was marked by an increase in the intensity of the abdominal pain, an unmeasurable blood pressure and a feeble pulse. Immediate paracentesis revealed 10 cc of fresh non coagulating blood. The diagnosis of ruptured ectopic pregnancy with abundant hemoperitoneum was considered and an emergency laparotomy with fluid and blood resuscitation was carried out. A midline laparotomy revealed a ruptured caesarean scar ectopic pregnancy with an abundant hemoperitoneum. Careful resection of the placenta and repair of the ruptured isthmic region of the uterus was carried out. Recovery after surgery was without complications and the patient was discharged on the 6th day following surgery.

**Conclusion:**

Caesarean scar pregnancy remains a very rare obstetric condition. Late diagnosis of this condition can be associated with serious life threatening obstetric complications. The rarity of the condition warrants a high index of suspicion among clinicians.

## Background

A caesarean scar pregnancy (CSP) is a very rare form of pregnancy that occurs when the developing blastocyst implants on a previous caesarean scar [1, 2]. Like all ectopic pregnancies, it can be potentially life threatening given the risk of heavy haemorrhage and uterine rupture [3, 4]. Although the number of reported cases seems to increase with the increasing rate of caesarean deliveries [1], the number of cases remain very few with a reported incidence ranging from 1 in 2216 to 1 in 1800 pregnancies [5].

Given its very rare nature, it constitutes a diagnostic dilemma among clinicians, especially in cases of late presentation with severe obstetric haemorrhage and in resource-limited settings where first trimester ultrasound scans are not routinely performed [6]. Failure to identify this condition on time could contribute to increase maternal morbi-mortality. A few cases have been reported in literature but none to our knowledge has been reported in Cameroon. We present a case of ruptured ectopic pregnancy at 20 weeks gestation on a successfully tried double caesarean section scar.

## Case presentation

A 29-year-old black Cameroonian of Bamileke ethnicity, gravida 5, para 3 with a past obstetric history of two consecutive caesarean sections done 9 and 5 years ago. Also noted was a successful trial of scar 2 years after the second caesarean section and a uterine evacuation following a miscarriage at 8 weeks of gestation.

The patient before consultation in our facility had been hospitalised twice in an integrated health centre for mild pelvic discomfort and two episodes of bleeding per vaginum, for which she was managed with injectable progesterone and discharged with favourable evolution.

The client was then received in our emergency department at 20 weeks gestation with mild to moderate intermittent lower abdominal pains associated to mild epigastralgia. The client however had a history of fever 24 h before consultation but no urgency, frequency nor mictalgia were reported. The parameters at entry had as temperature 37 °C, blood pressure of 110/60 mmHg, Pulse rate of 94 beats per minute. Other physical examination findings included mild generalised abdominal tenderness on superficial and deep palpation which was worse at the pelvic region. The provisional diagnosis of threatened abortion (due to malaria or asymptomatic bacteriuria was considered) with acute appendicitis in pregnancy as differential. A rapid diagnostic test for malaria was done which was negative. The team on duty directly went into management with Omeprazole 20 mg tablets (1 tablet 12 hourly), phloroglucinol 80 mg injectable (1 ampoule 8 hourly) and Ampicilline injectable (1 g 8 hourly) while thick blood smears, urinalysis, obstetric ultrasound, and full blood count were requested for the next morning.

Her evolution 4 h later was marked by an increase in the intensity of the abdominal pain which became generalised with altered general condition. The blood pressure was unmeasurable and the pulse feeble. Immediate paracentesis revealed 10 cc of fresh non coagulating blood. The diagnosis of ruptured ectopic pregnancy with abundant hemoperitoneum was considered and because of hemodynamic instability, the patient was immediately prepared for an emergency laparotomy with fluid and blood resuscitation.

**Fig. 1:**
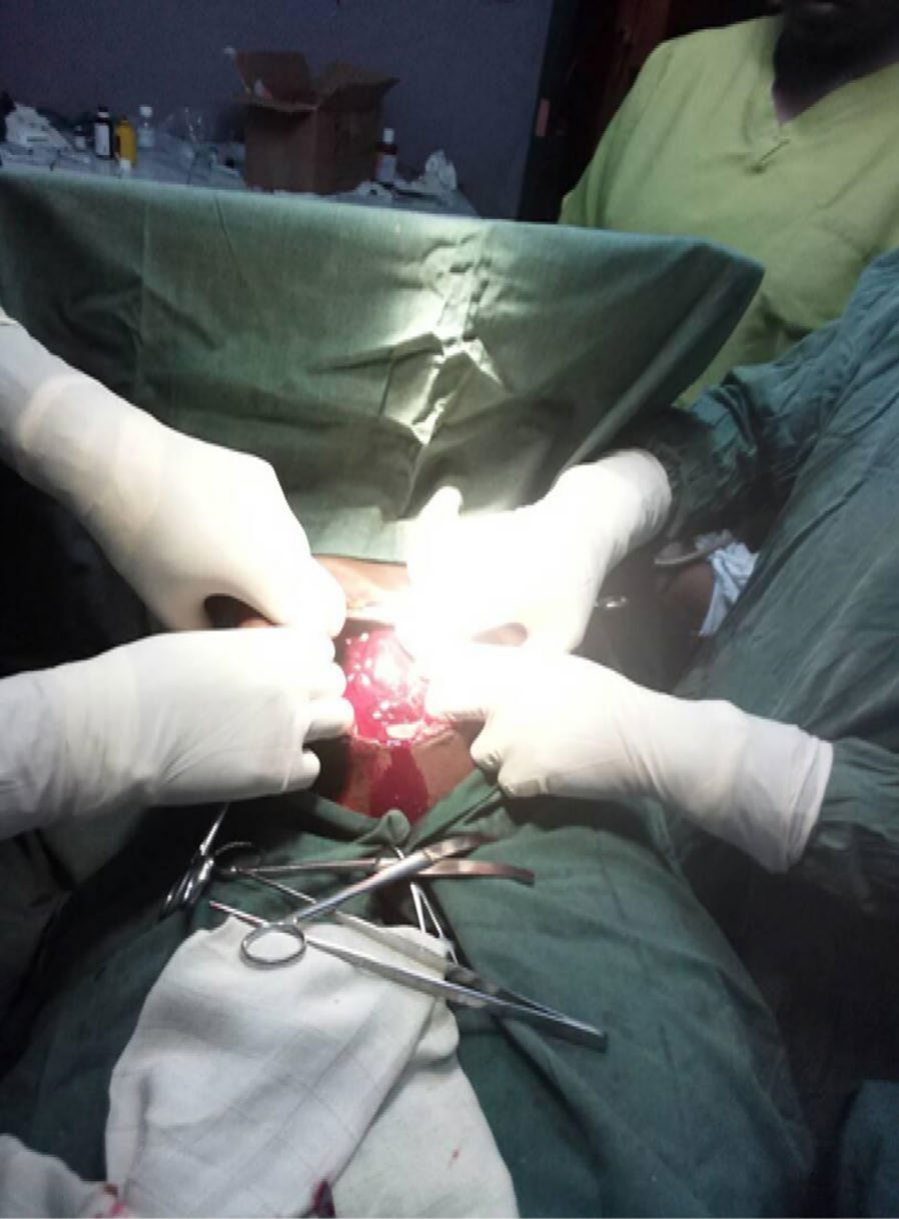
Image showing midline laparotomy and hemoperitoneum

**Fig. 2:**
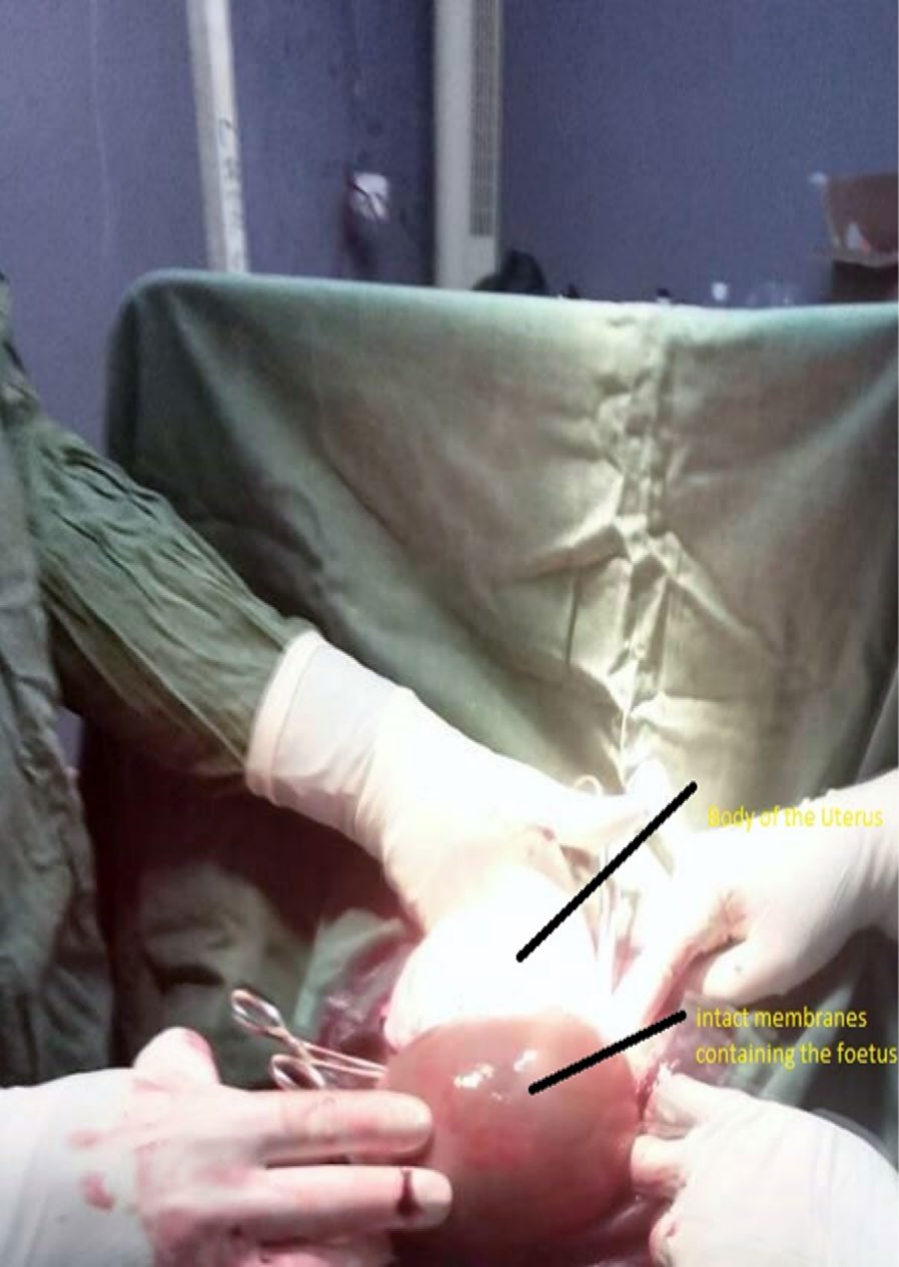
Image showing the uterus and the intact membranes

**Fig. 3:**
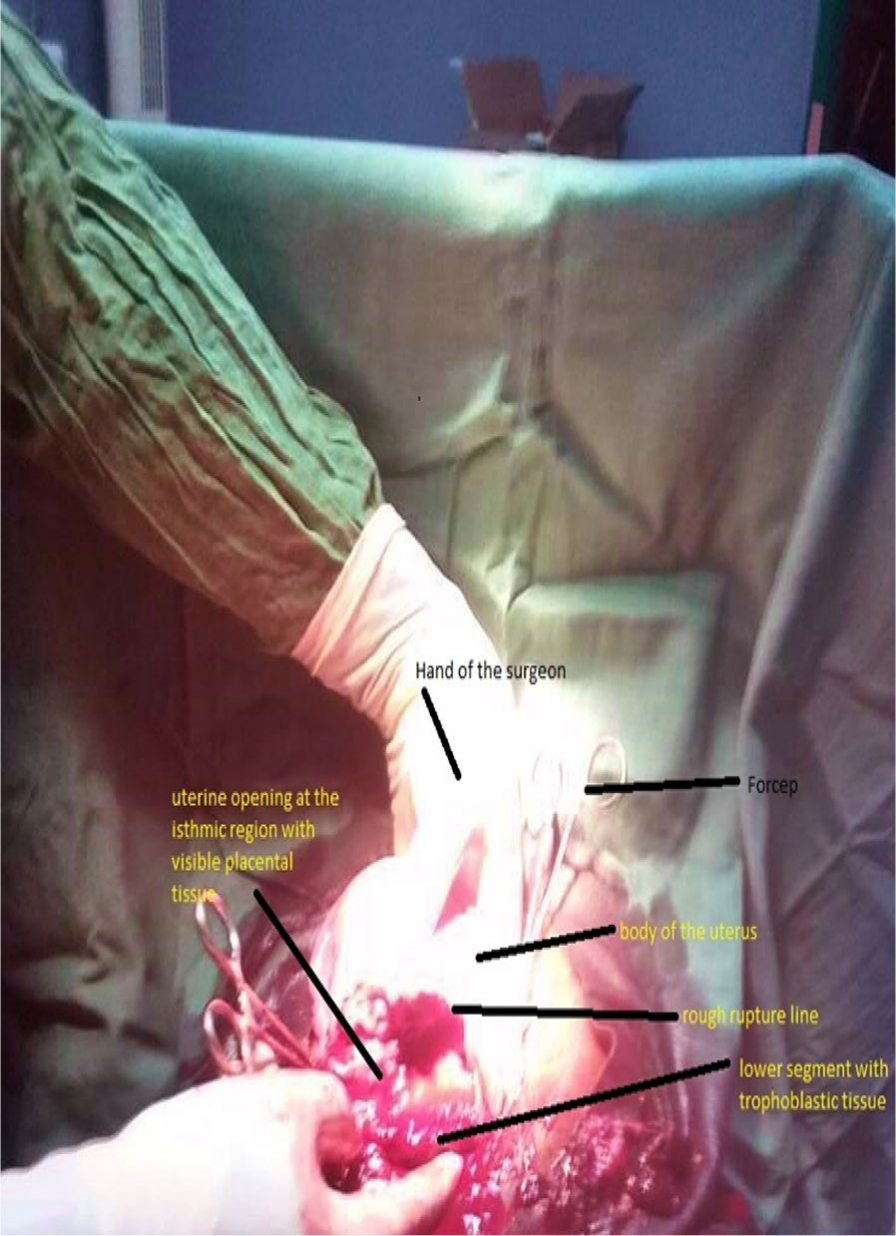
Image showing the rupture line along the old caesarean scar

A midline subumbilical laparotomy (Fig. 1) was carried under general anaesthesia. The perioperative findings included: an abundant hemoperitoneum (Fig. 1) estimated to two litres; intact membranes containing the foetus in the abdominal cavity; rupture line along the old caesarean scar at the isthmic uterine region, detached placental tissue most of which was still inserted and covering the internal cervical os (see Figs. 2 and 3). The uterus and the intact membranes were carefully exteriorised. The hemoperitoneum was reduced with the help of sterile abdominal towels. Careful detachment of the placenta and repair of the opening with vicryl 1 suture was done. The patient was placed on antibioprophylaxis with Ampicilline injectable (1 g 8 hourly for 2 days) and analgesics (1000 mg of injectable paracetamol 8 hourly for 3 days). The postoperative recovery was uneventful and the patient was discharged 7 days following surgery. Follow-up of the patient to 1 year after surgery was uneventful.

## Discussions and conclusions

Caesarean scar pregnancy (CSP) is the rarest form of ectopic pregnancy and can be associated to serious and life threatening obstetric complications. The incidence of CSP varies from 1 in 1800 to 1 in 2226 pregnancies [1] and accounts for 6% of ectopic pregnancies in women with prior cesarean deliveries [7]. Given its location and possibility of growth with associated normally increasing titres of beta-hCG, a high suspicion index remains the only efficient way to identify and manage this life threatening obstetric condition on time. Even though the main cause of CSP is not known, in literature, it has been associated to history of uterine trauma, caesarean section [8], invitro fertilization, manual placental removal, adenomyosis and myomectomy [1, 5, 9].

In most cases of CSP, the gestation sac is usually completely surrounded by myometrium and the fibrous tissue of the scar, quite separate from the endometrial cavity [1]. These forms are generally termed “intramural pregnancies” (that is, completely confined to the myometrium) as opposed to the other forms which develop without total confinement to the myometrium [10]. The most efficient and readily available tool for early diagnosis is transvaginal ultrasound. Suggested sonographic criteria to be considered when making diagnosis of CSP in literature include: empty uterine cavity; location of trophoblast mainly between bladder and anterior uterine wall; thin or absent layer of myometrium between the gestational sac and the bladder; identification of a discontinuity in the anterior wall of the uterus on a sagittal view running through the amniotic sac and an empty endo-cervical canal [10, 11]. No classical treatment methods have been described in literature but treatment methods range from close monitoring to viability and term to conservative and radical surgery in case of uterine rupture [2, 12].

In our case, the patient had undergone two caesarean sections with a successful trial of the two scars and a dilatation and curettage for an incomplete abortion. The trial of the double scar and the subsequent dilatation and curettage might have compromised the integrity of the caesarean scar thereby exposing her to CSP. In CSP, it is thought that implantation villi find their way into the myometrium through a microtubular tract between the caesarean section scar and the endometrial canal [1]. The placenta and the conceptus were totally expulsed from the uterine opening with an intact posterior uterine wall.

Most cases of CSP are usually diagnosed in the first trimester. Patients are generally amenorrheic women with painless vaginal bleeding early in pregnancy (39%), mild abdominal pain or discomfort (16%) [1, 5]. In case of rupture, patients will present with severe acute pain of sudden onset and profuse bleeding and hypovolemic shock. However, 9% of patients with non-ruptured forms might present just with abdominal pains and may be asymptomatic with incidental diagnosis in 37% of cases [1]. This constitutes the diagnostic dilemma as none of these signs directly point to caesarean scar pregnancy as the closest possible diagnosis. This therefore calls on a high index of suspicion of CSP especially in women presenting with any of the above symptoms and any of the risk factors. On suspicion of CSP radiological evaluation is mandatory [4]. In most cases without rupture or onset of rupture, clinical examination is usually unremarkable. The uterus and the abdomen are usually tender in case of rupture [9]. Failure of diagnosis and extension into the second trimester is usually associated with a higher risk of rupture and haemorrhage. It was the case with the client as the diagnosis was only made when the life of the patient was seriously threatened.

In the above presented case, our patient had presented twice with mild pervaginal bleeding in the first trimester which was managed with injectable progesterone. No first trimester ultrasound was done by the patient because of financial barriers. The influence of the low income setting here on the health of the patient is clear. This is a cause for concern as most pregnant women in our setting start antenatal consultations only in the second trimester and the ultrasounds are done only in the late second trimester. The very rare nature of ectopic pregnancies in the developed world has also been associated to missed and mismanaged cases of CSP even when first trimester ultrasound scans are readily available [11]. Early diagnosis and management of this life threatening condition is therefore even more impeded in a resource-limited setting given that the access of pregnant women to ultrasound scans is still limited.

The working diagnosis (threatened abortion due to malaria or asymptomatic bacteriuria with acute appendicitis in pregnancy as differential) was considered given the very low suspicion index of CSP within the team. This low suspicion index and the absence of the ultrasonographer allowed for progression of the condition to its severe and life threatening stage. In case of rupture, medical treatment with methotrexate or curettage is not a suitable option. Emergency laparotomy with resection of the ectopic and repair of the uterus or hysterectomy are suitable options. In our case, given the future fertility desire expressed by the couple and peroperative findings, we completed resection of the ectopic and repaired the uterine opening.

Caesarean scar pregnancy remains a very rare obstetric condition. Late diagnosis of this condition can be associated with serious life threatening obstetric complications. Given the rare nature ectopic pregnancies and more specifically CSP, clinicians should have a high index of suspicion especially in amenorrheic women with risk factors. First trimester ultrasonography remains indispensable in the appropriate screening for such conditions especially in clients presenting with risk factors. Improving access to prenatal services (while emphasizing on the need to take up these services early in pregnancy and systematically meeting competent ultrasonographers in the first trimester especially in patients with risk factors for CSP) could go a long way to improve the condition and the prognosis of patients with CSP.

## Abbreviation

CSP: caesarean scar pregnancy
